# Histone Acetyltransferase SlGCN5 Regulates Shoot Meristem and Flower Development in *Solanum lycopersicum*

**DOI:** 10.3389/fpls.2021.805879

**Published:** 2022-01-21

**Authors:** Amangul Hawar, Shiqi Xiong, Zhen Yang, Bo Sun

**Affiliations:** State Key Laboratory of Pharmaceutical Biotechnology, School of Life Sciences, Nanjing University, Nanjing, China

**Keywords:** tomato, SAM, *SlGCN5*, *SlWUS*, *SlADA2*

## Abstract

The histone acetyltransferase (HAT) general control non-repressed protein 5 (GCN5) plays important roles in plant development *via* epigenetic regulation of its target genes. However, the role of GCN5 in tomato, especially in the regulation of tomato shoot meristem and flower development, has not been well-understood. In this study, we found that silencing of *Solanum lycopersicum GCN5* (*SlGCN5*, *Solyc10g045400.1.1*) by virus-induced gene silencing (VIGS) and RNA interference (RNAi) resulted in the loss of shoot apical dominance, reduced shoot apical meristem (SAM) size, and dwarf and bushy plant phenotype. Besides, we occasionally observed extra carpelloid stamens and carpels fused with stamens at the late stages of flower development. Through gene expression analysis, we noticed that SlGCN5 could enhance *SlWUS* transcript levels in both SAM and floral meristem (FM). Similar to the known function of GCN5 in *Arabidopsis*, we demonstrated that SIGCN5 may form a HAT unit with *S. lycopersicum* alteration/deficiency in activation 2a (SlADA2a) and SlADA2b proteins in tomato. Therefore, our results provide insights in the SlGCN5-mediated regulation of SAM maintenance and floral development in tomato.

## Introduction

Plants have a unique ability to give rise to new organs continuously due to the indeterminate production of undifferentiated stem cells located in specific regions of meristems. The shoot apical meristem (SAM) gives rise to the aerial organs, and the maintenance of SAM is key for the development of plants and adaptation to the changes of external environment (Pfeiffer et al., 2017). Unlike *Arabidopsis*, tomato is a typical sympodial plant. After the formation of 8–10 leaves, tomato SAM terminated and transforms into inflorescence meristem (IM) and sympodial meristem (SYM), which are formed at the leaf axils beneath the IM to sustain continuous growth. Thereafter, IM transforms to floral meristem (FM) and initiates a second IM in the meantime (Schmitz and Theres, 1999; Périlleux et al., 2014). Tomato FMs generate four whorls of floral organs, namely, sepals, petals, stamens, and carpels, sequentially in concentric whorls (Sekhar and Sawhney, 1984).

In *Arabidopsis*, the maintenance of the stem cell pool in the SAM is regulated by *CLAVATA-WUSCHEL* (*CLV-WUS*) feedback loop (Schoof et al., 2000). In this feedback loop, WUS could directly induce stem cell identity and the expression of the stem cell marker gene *CLV3* (Yadav et al., 2011; Daum et al., 2014). The *CLV* genes including *CLV1* and *CLV3* repress *WUS* through signaling cascades (Shang et al., 2019; Han et al., 2020), therefore coordinating and balancing stem cell proliferation with differentiation. The *CLV-WUS* feedback loop appears to be highly conserved across different plant species (Somssich et al., 2016). In tomato, the mutation of *SlCLV3* promotes stem cell overproliferation and results in extra floral organs and bigger fruits (Rodríguez-Leal et al., 2017). In *SlWUS* RNA interference (RNAi) lines, plants have reduced flower size and fruit locule numbers (Li et al., 2017). Changes in tomato meristem size have also been observed in *fasciated* (*fas*) and *locule number* (*lc*) mutants, both of which have misexpression of *SlWUS* and *SlCLV3*, respectively (Muños et al., 2011; Xu et al., 2015; Chu et al., 2019).

In various plant species, studies have discovered that the *CLV-WUS* regulatory loop could be modified by many additional factors, which can contribute to plant growth and productivity (Galli and Gallavotti, 2016). Among these factors, histone modifications including acetylation or methylation on several lysine residues of H3 are important for gene expression during plant development (Servet et al., 2010). Histone acetyltransferases (HATs) can catalyze acetylation of specific lysine residues on histone N-tails and leads to transcriptional regulation (Bannister and Kouzarides, 2011). It has been reported that in most cases, GCN5 acts as the catalytic core of the HAT complex, which also include vital adaptor proteins ADA2a and ADA2b (Shahbazian and Grunstein, 2007). GCN5 acetylates lysine 14 of histone H3 (H3K14ac) and influences H3K9ac and H3K27ac levels in promoter region of its targets (Benhamed et al., 2006; Servet et al., 2010; Ruggieri et al., 2020). In contrast, ADA2 proteins could help increase the HAT activity of GCN5 (Mao et al., 2006).

In *Arabidopsis*, both GCN5 and ADA2b are required for many developmental processes such as shoot apical dominance, root meristem activity, leaf development, IM or FM function, and flower fertility (Bertrand et al., 2003; Vlachonasios et al., 2003; Cohen et al., 2009; Kornet and Scheres, 2009; Anzola et al., 2010; Servet et al., 2010). In poplar trees, ABRE-motif binding protein PtrAREB1-2 binds to *PtrNAC* genes, recruits the HAT unit ADA2b-GCN5 by forming a AREB1-ADA2b-GCN5 protein complexes, and results in increased H3K9 acetylation levels on *PtrNAC* genes (Li et al., 2019). In rice, the homeodomain protein OsWOX11 recruits a HAT complex containing OsGCN5 to establish the programs of cell proliferation in crown root meristem (Zhou et al., 2017). One study implies that the SAGA (Spt-Ada-GCN5 acetyltransferase) complex is an evolutionarily conserved complex that has a critical role in various developmental processes (Spedale et al., 2012).

In this work, we identified *SlGCN5*, *SIADA2a*, and *SlADA2b* in tomato and found that SlGCN5 can form a HAT unit with SlADA2a and SlADA2b and influences H3K9ac, H3K14ac, and H3ac at the genomic level. Silencing of *SlGCN5* resulted in dwarf plant phenotype, reduced SAM size, carpelloid stamens, and fusion of carpels with stamens in flowers. Furthermore, we proposed that SlGCN5 could enhance *SlWUS* expression, thereby maintaining stem cell homeostasis in tomato.

## Materials and Methods

### Plant Materials and Growth Conditions

*Arabidopsis* plants and wild-type (WT) tomato (*Solanum lycopersicum*) plants of Micro-Tom (MT) and transgenic *Arabidopsis* and tomato lines were grown in the greenhouse, under long-day condition (16-h light/8-h dark). For transformation, tomato cotyledons were cultivated *in vitro* in MS medium in a growth chamber (Panasonic, MLR-352H-PC) at 22°C/20°C under16-h light and 8-h dark conditions.

### Construction of TRV-*SlGCN5* and RNAi Vectors and Tomato Transformation

The tobacco rattle virus (TRV)-based vectors, i.e., pTRV1 and pTRV2, were used for virus-induced gene silencing (VIGS). To construct a pTRV2-*SlGCN5* vector, according to the website^1^, a 400-bp DNA fragment of the *SlGCN5* CDS was amplified from tomato cDNA using primers in Supplementary Table 1. The constructs were introduced into *Agrobacterium tumefaciens* GV301. Then, VIGS assays were carried out as previously described (Fu et al., 2005).

To generate amiRNA for silencing *SlGCN5*, the amiRNAs (21-nt) were designed by using the web MicroRNA Designer (WMD3^2^). Pre-amiRNA was assembled by several rounds of PCR using primers listed in Supplementary Table 1. The final PCR fragments were driven under 35S promoters in pCHF3 vector. After *SlGCN5-*RNAi construct is transformed into *Agrobacterium* GV3101, the *Agrobacterium*-mediated transformation of tomato cotyledons was performed as described (Cortina and Culiáñez-Macià, 2004; Tripodi, 2020).

### Phylogenetic Analysis

For phylogenetic analysis, the coding sequences of *ADA2* orthologs were retrieved from JGI Genome Portal and Resources for Plant Comparative Genomics^3^ by BLAST using *AtADA2a* coding sequence as a query with default parameters. The phylogenetic tree of ADA2 orthologs in dicots was constructed by W-IQ-TREE (Nguyen et al., 2015), which identified the best evolutionary model as the general time reversible model (GTR + F + I + G4). The non-parametric UltraFast Bootstrap (UFBoot) method (Minh et al., 2013) was used to calculate the node support, and 1,000 bootstrap pseudo replicates were performed with bootstrap values indicated in branches.

### Subcellular Localization Analysis

DNA fragment of *SlGCN5* was amplified by PCR (primers are listed in Supplementary Table 1) and inserted into pGreenII vector to generate the SlGCN5-GFP (green fluorescent protein) fusion protein. Then, pGreenII vector-based 35S:SlGCN5-GFP and the control vector pGreenII-based 35S:GFP were transformed into *A. tumefaciens* strain GV3101 and injected into 4-week-old tobacco leaves. GFP fluorescence was observed using Olympus (BX53) microscope after 72 h of infiltration.

### RNA Extraction and Expression Analyses

RNA extraction and quantitative real-time (qRT)-PCR analysis were carried out as described previously (Sun et al., 2019). *ACTIN2* and *SlACTIN2* were served as the internal control in *Arabidopsis* and tomato, respectively. The sequences of all primers are listed in Supplementary Table 1.

### *In situ* Hybridization

RNA *in situ* hybridization was performed as described previously (Sun et al., 2019). Briefly, *SlGCN5* (*Solyc10g045400.1.1*) and *SlWUS* (*Solyc02g083950*) probes were synthesized from cDNA by using the primers listed in Supplementary Table 1, and the PCR products were cloned into pGEM-T Easy vector (TIANGEN, VT307). After linearization, the DIG RNA labeling kit (Roche, 11175025910) was used for *in vitro* transcription of probes. The experiments were performed twice using two different batches of plants. Photographs were taken by using an Olympus BX53 microscope.

### Yeast Two-Hybrid Assay

To obtain yeast two-hybrid vectors, the full-length *SlGCN5* was cloned into pGADT7 (Clontech). The full-length *SlADA2a* and *SlADA2b* were individually cloned into pGBKT7 (Clontech). The yeast two-hybrid assay was performed using the Yeastmaker Yeast Transformation System 2 (Clontech, T2001) according to the instruction of the manufacturer. Primer sequences are provided in Supplementary Table 1.

### Bimolecular Fluorescence Complementation Assay

For bimolecular fluorescence complementation (BiFC) assay, SlGCN5 and SlADA2 were tagged with the C-terminal part of YFP (YFPC) and the N-terminal part of YFP (YFPN), respectively, as previously described (Kudla and Bock, 2016). Cloning primers are listed in Supplementary Table 1. After vectors were transformed into *Agrobacterium*, the *Agrobacterium* carrying different vectors were co-infiltrated into tobacco (*Nicotiana benthamiana*) leaves of 4-week-old plants as described previously (Sparkes et al., 2006). The infected tobacco leaves were cultured for 72 h before observation. Notably, 5 μg/ml DAPI was used to visualize the nuclei. The fluorescence was observed by using Olympus (BX53) microscope.

### Statistical Analysis

The statistical analysis was conducted using two-tailed *t*-test. The statistically significant differences are indicated by *p < 0.05, ^**^p < 0.01, or ^***^p < 0.001.

## Results

### Silencing of *SlGCN5* Affects Tomato Plant Development

**FIGURE 1:**
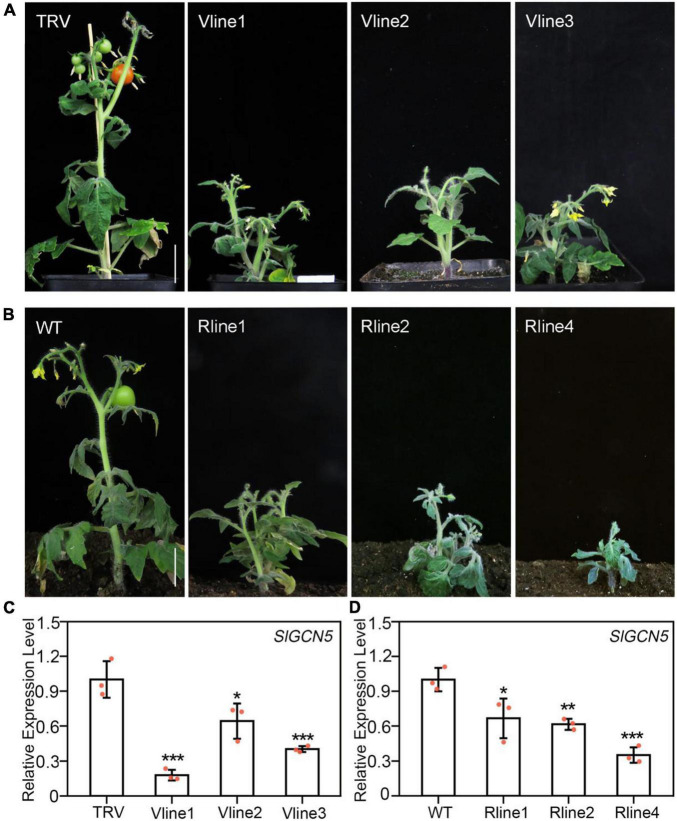
Silencing of *SlGCN5* affects plant development. **(A)** Phenotype of three independent TRV-*SlGCN5* lines of Micro-Tom tomato. V lines represent *SlGCN5-VIGS* lines. **(B)** Phenotype of three independent *SlGCN5*-*RNAi* lines. **(C)** qRT-PCR analysis of *SlGCN5* transcripts in TRV control and TRV-*SlGCN5* plants. **(D)** Transcript levels of *SlGCN5* in *SlGCN5*-*RNAi* lines relative to wild type (WT). Error bars represent SD of three biological replicates. Asterisks indicate significant differences (*p < 0.05, **p < 0.01, and ***p < 0.001). Scale bars = 3 cm.

GCN5 was reported to participate in many biological processes in *Arabidopsis*, especially in plant development (Vlachonasios et al., 2003). In this study, we aimed to investigate the function of GCN5 in tomato development. For this purpose, we first searched for putative homologs of *AtGCN5* in tomato genome sequence, and only one homologous gene with three isoforms was identified (Supplementary Figure 1). Among these three isoforms, we chose the one with the highest expression level in tomato inflorescences and the highest protein similarity with AtGCN5 for further study. To explore the effect of *SlGCN5* silencing, tomato seedlings in two-cotyledon stage were infected with *Agrobacterium* carrying the TRV-based VIGS of *SlGCN5* vector. TRV-*SlGCN5* plants exhibited predominantly developmental defects, including reduced plant height, loss of shoot apical dominance, altered pattern of axillary shoot development, shortened internode, late flowering, and male sterility (Figure 1A), suggesting that SlGCN5 is required in various tomato plant developmental processes. To verify the phenotype of TRV-*SlGCN5*, we created *SlGCN5-RNAi* plants and found all three of the *SlGCN5-RNAi* lines exhibited similar phenotype with TRV-*SlGCN5* plants (Figure 1B).

Results of qRT-PCR showed that *SlGCN5* transcription level in the TRV-*SlGCN5*-infected plants was significantly lower than plants infected with TRV control (Figure 1C), confirming that the abnormal phenotypes are caused by *SlGCN5* gene silencing. Similarly, the expression level of *SlGCN5* was significantly reduced in the RNAi lines compared with WT plants (Figure 1D). Due to the similar phenotypes of *SlGCN5*-*RNA*i and TRV-*SlGCN5* plants, we used TRV-*SlGCN5* plants for subsequent functional studies in tomato plant development.

### SlGCN5 Is Located in the Nucleus and Highly Expresses in Tomato Early Floral Bud

**FIGURE 2:**
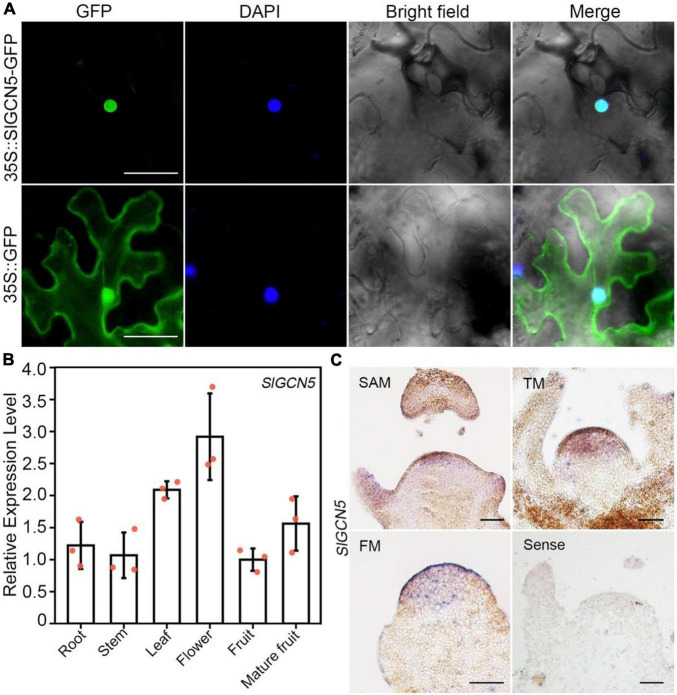
Subcellular localization and gene expression pattern of *SlGCN5*. **(A)** Subcellular localization of SlGCN5 in nuclei. 35S:SlGCN5-GFP represents SlGCN5-GFP fusion protein. 35S:GFP represents the control. Scale bars = 20 μm. **(B)** qRT-PCR analysis of *SlGCN5* in different tomato organs. *SlACTIN* served as the internal control. Error bars represent SD of three biological replicates. **(C)** *In situ* hybridization of *SlGCN5* in SAM, floral transition meristem (TM), and FM, respectively. Scale bars = 50 μm.

To investigate the expression pattern of *SlGCN5*, we first analyzed subcellular localization of SlGCN5 protein. Results showed that SlGCN5-GFP fusion protein driven by constitutive cauliflower mosaic virus 35S promoter exclusively localized in the nucleus (Figure 2A), suggesting that SlGCN5 may have a putative role in histone modification. During tomato plant development, *SlGCN5* transcripts expressed widely in roots, stems, leaves, flowers, and fruits (Figure 2B). Our *in situ* hybridization assays revealed that *SlGCN5* is strongly expressed in the upper cell layers of SAM. Meanwhile, *SlGCN5* was expressed throughout the entire floral transition meristem and FM of WT plants, which may overlap with the expression domain of *SlWUS* (Figure 2C), hinting at a potential role for SlGCN5 in regulation of meristematic activities.

### SlGCN5 Catalyzes Histone Acetylation

**FIGURE 3:**
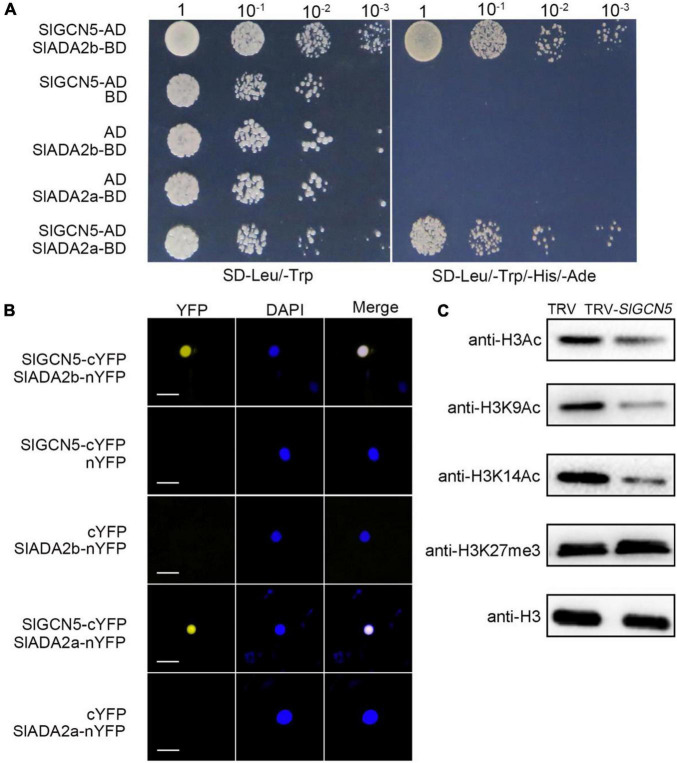
SlGCN5 functions as a histone H3 acetyltransferase. **(A)** Yeast two-hybrid assays of SlGCN5, SlADA2a, and SlADA2b. Full-length cDNAs of *SlGCN5*, *SlADA2a*, and *SlADA2b* were cloned into AD (the prey plasmid pGADT7) and BD (the bait plasmid pGBKT7), respectively. Yeast cells transformed with the indicated plasmids were grown on medium lacking leucine and tryptophan (SD/-Leu/-Trp) and selective medium lacking leucine, tryptophan, histidine, and adenine (SD/-Leu/-Trp/-His/-Ade). **(B)** Bimolecular fluorescent complementation analysis in tobacco leaves. Merge refers to merged images for yellow fluorescent protein (YFP) and DAPI fluorescence. SlGCN5 and SlADA2a/b were fused to cCFP and nYFP, respectively. Scale bars = 20 μm. **(C)** Histone acetyltransferase activity of SlGCN5 determined by *in vivo* histone acetyltransferase assay. Histone acetylation levels were detected by immunoblotting with antibodies of the indicated histone acetylation marks in TRV and TRV-*SlGCN5* plants. Anti-H3 antibody was used as loading control.

The SAGA (Spt-Ada-Gcn5 acetyltransferase) complex is highly conserved for active regulation of gene transcription in yeast and plants (Carrozza et al., 2003; Vlachonasios et al., 2003; Zhou et al., 2017; Li et al., 2019). We also identified ADA2a- and ADA2b-like proteins in tomato (Supplementary Figure 2) and named them as SlADA2a and SlADA2b, respectively, which have the highest homology with AtADA2a and AtADA2b in *Arabidopsis*. SlADA2a and SlADA2b have 3 and 2 isoforms respectively. According to the transcript analysis results in tomato inflorescences, XP_004243566 and XP_004239816 were selected as representatives of *SlADA2a* and *SlADA2b* for further study (Supplementary Figure 3). To confirm the interactions between SlADA2a with SlGCN5 and SlADA2b with SlGCN5, we cloned the full-length cDNAs of SlADA2a, SlADA2b, and SlGCN5 and performed yeast two-hybrid assays. The results showed that SlGCN5 can interact with both SlADA2a and SlADA2b in yeast cells (Figure 3A). To verify the yeast two-hybrid results, we performed BiFC analysis in tobacco (*Nicotiana tabacum*) leaves. SlGCN5 was fused to the C-terminus of YFP and named as SlGCN5-cYFP. SlADA2a or SlADA2b was fused to the N-terminus of YFP and named as SlADA2a-nYFP or SlADA2b-nYFP, respectively. We noticed interactions between SlGCN5 and SlADA2a, as well as SlGCN5 and SlADA2b in the nucleus, both of which gave clear signals (Figure 3B). These results suggest that SlGCN5 can interact with both SlADA2a and SlADA2b and that the three proteins may form a protein complex.

To test the HAT activity of SlGCN5 *in vivo*, we compared histone acetylation levels in TRV-*SlGCN5* plants with TRV control plants by immunoblotting, using anti-H3K9Ac, anti-H3K14Ac, anti-H3Ac, and anti-H3K27me3 antibodies. Our results revealed that obvious reduction of H3ac, H3K9ac, and H3K14ac levels in TRV-*SlGCN5* compared with the TRV control plants (Figure 3C), suggesting that SlGCN5 can catalyze acetylation on histone H3, specifically at H3K9 and H3K14 residues. These results are consistent with the known function of AtGCN5, which was reported to catalyze H3K14ac, and additional histone residues, including H3K9, H3K18, H3K27, and H3K36, and other histones such as H4 and H2B in *Arabidopsis* (Kuo et al., 1996; Grant et al., 1997; Morris et al., 2007). To confirm the role of *SlGCN5* in plant development, we generated transgenic *Arabidopsis* plants by transforming the null-mutant *gcn5-7* with *35S:SlGCN5-GFP*. *35S:SlGCN5-GFP gcn5-7* plants have noticeable gene and protein expressions of SlGCN5, which are examined by qRT-PCR and Western blot (Supplementary Figure 4A). Furthermore, *35S:SlGCN5-GFP gcn5-7* plants show almost fully rescued phenotype compared with *gcn5-7* (Supplementary Figure 4B), indicating that SlGCN5 functions similarly as AtGCN5.

### SlGCN5 Regulates Tomato Shoot Meristem and Flower Development

**FIGURE 4:**
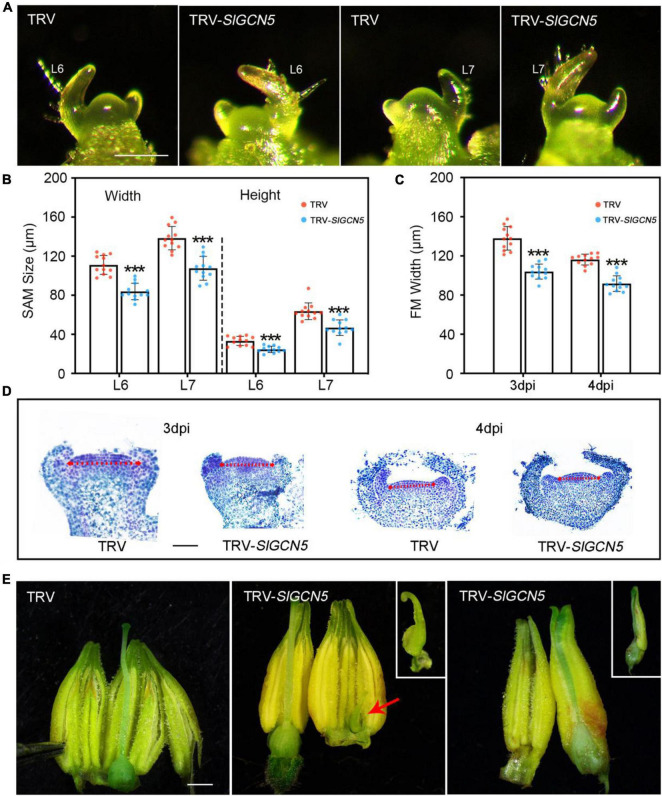
SAM and floral phenotype of silenced-*SlGCN5* plants. **(A)** Images of the SAMs from TRV control and TRV-*SlGCN5* plants. L6 and L7 indicate Leaf 6 and Leaf 7, respectively. Scale bars = 200 μm. **(B)** SAM size from TRV control and TRV-*SlGCN5* plants. Error bar indicates SD of 12 biological replicates. **(C)** FM size from TRV control and TRV-*SlGCN5* plants at 3 and 4 dpi (days post floral initiation). Error bar indicates SD of 12 biological replicates. **(D)** Longitudinal sections of floral meristem of TRV control and TRV-*SlGCN5* plants. The red dash arrow marks the width of each floral meristem. Scale bars = 50 μm. **(E)** Flowers of TRV and TRV-*SlGCN5*. Scale bars = 1 mm. Asterisks indicate significant differences between TRV control and *TRV-SlGCN5* (***p < 0.001).

*SlGCN5*-silenced plants exhibited reduced plant height. Thus, we measured SAM size in TRV-*SlGCN5* and TRV control plants and observed reduced SAM size in TRV-*SlGCN5* at different developmental stages compared with TRV control plants (Figures 4A,B). We also observed reduced FM width but relatively unchanged FM height (Supplementary Figure 5) in TRV-*SlGCN5* young floral buds prior to the emergence of the carpel primordia (Figures 4C,D). Although FM size in TRV-*SlGCN5* is reduced, floral organ number remains largely unaffected. However, in TRV-*SlGCN5* flowers, we occasionally noticed some carpelloid stamens and carpels fused with stamens [2/15 (13.3%) independent transgenic lines show abnormal flowers] (Figure 4E). These results implied that silencing of *SlGCN5* resulted in reduced SAM and FM sizes in tomato and may also influence reproductive floral organ development.

### SlGCN5 Positively Regulates *SlWUS* Expression

**FIGURE 5:**
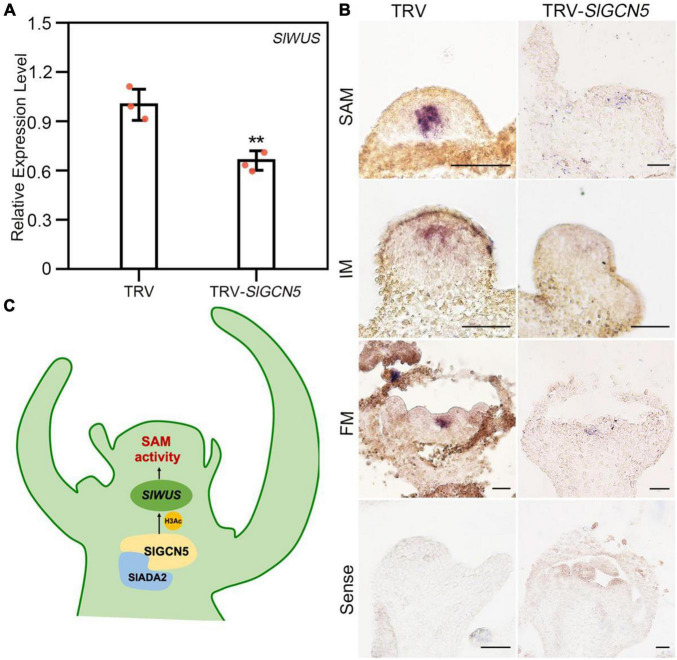
SlGCN5 positively regulates *SlWUS* in tomato. **(A)** qRT-PCR analysis of *SlWUS* expression level in SAM. The error bar represents SD of three biological replicates. The asterisks indicate significant differences between TRV and TRV-*SlGCN5* (**p < 0.01). **(B)** Expression of *SlWUS* mRNA in TRV control and TRV-*SlGCN5* plants by *in situ* hybridization. Scale bars = 50 μm. **(C)** Model: SlGCN5 together with SlADA2a and SlADA2b could form HAT complex, which positively regulate *SlWUS* to ensure the proper development of SAM.

The reduced SAM and FM size leads us to examine expression changes of *SlWUS* in TRV-*SlGCN5* plants. Expression analysis by qRT-PCR revealed that *SlWUS* transcript level was significantly reduced in TRV-*SlGCN5* meristems (Figure 5A). To validate the qRT-PCR results, expression pattern of *SlWUS* was examined by *in situ* hybridization assays. We noticed obviously reduced expression of *SlWUS* mRNA in TRV-*SlGCN5* SAMs and FMs (Figure 5B) compared with TRV control plants. These results suggested that SlGCN5 may positively regulate *SlWUS* expression in tomato shoot meristem and FM. Furthermore, we observed remarkable decrease in the transcript level of *SlCLV1* and *SlCLV3*, the other two key factors in *CLV-WUS* feedback loop, in TRV-*SlGCN5* meristems by qRT-PCR analysis (Supplementary Figure 6). These results indicate that SlGCN5 may potentially regulate multiple genes in meristem development of tomato.

## Discussion

Histone lysine acetylation is an essential chromatin modification for epigenetic regulation of gene expression in plant development and plant response to environmental stress. AtGCN5 was identified as the first transcription-linked HAT (Brownell et al., 1996), with specificity for histone H3K14ac (Kuo et al., 1996). In addition, GCN5 could also acetylate histone lysine residues such as H3K9, H3K18, H3K23, H3K27, and H3K36 and other histones such as H4 and H2B (Grant et al., 1997; Morris et al., 2007). The SAGA complex is an evolutionarily conserved HAT complex (Spedale et al., 2012), which catalyzes histone acetylation for modulating gene expression and participates in various developmental processes in eukaryotes. In this study, we showed that SlGCN5 can acetylate histones H3K9 and H3K14 at the genomic level in tomato, and SlGCN5 also interacts with SlADA2a and SlADA2b to form HAT unit.

Shoot apical meristem is an organized structure and responds to different development signals. The stem cell pool is maintained within the central zone of the SAM (Fletcher, 2018). Compromised SAM activity leads to premature plant growth stagnation before forming full organs (Laux et al., 1996; Kieffer et al., 2006), whereas plants with overproliferated stem cells in SAM can produce many extra organs (Clark et al., 1993; Taguchi-Shiobara et al., 2001; Yuste-Lisbona et al., 2020). Therefore, the maintenance of SAM homeostasis is key for plant development. It is well-understood that conserved *CLV-WUS* feedback signaling is important for the maintenance of SAM activity (Somssich et al., 2016), but it is not well-known how this feedback loop is modified in various plant species. In this study, we characterized the function of SlGCN5 and studied its role in SAM maintenance. Our data indicate that *SlGCN5* is important to maintain SAM activity in tomato. Weakened SlGCN5 activity affects SAM development and resulted in reduced SAM and FM size (Figure 4). Consistent with the phenotype, we also observed reduced *SlWUS* expression (Figure 5B) in SAM and FM in the plants with compromised *SlGCN5* activity. However, we did not observe obvious changes in floral organ numbers. Instead, we occasionally observed some carpelloid stamens and carpels fused with stamens (Figure 4E). These phenotypes resemble the *S. lycopersicum GT11 (SlGT11)* mutant, in which the function of floral B-class genes was affected (Yang et al., 2020). Therefore, we suspect that the transformation of floral homeotic genes may also exist in TRV-*SlGCN5* plants and that *SlGCN5* could participate in the maintenance of floral organ identity.

Modulation of *CLV-WUS* pathway is one important approach to increase crop yield (Fletcher, 2018). In tomato, several transcription factors that could influence the *CLV-WUS* loop also have been discovered. DEFECTIVE TOMATO MERISTEM (DTM) forms a negative feedback loop with the class III homeodomain-leucine zipper (HD-ZIP III) transcription factors to confine *SlCLV3* and *SlWUS* expression to specific domains in the shoot meristem of tomato (Xu et al., 2019). APETALA2/ethylene responsive factor (AP2/ERF) superfamily transcription factor excessive number of floral organs (ENO) regulates *SlWUS* expression to restrict stem cell proliferation, thereby maintaining floral stem cell homeostasis (Yuste-Lisbona et al., 2020). In addition to transcription factors, *SlWUS* expression can also be regulated by chromatin remodeling factors such as histone deacetylase 19 in tomato (Bollier et al., 2018).

In this study, we identified and investigated the function of *SlGCN5* in tomato meristem development and found that SlGCN5 acts as an acetyltransferase to activate the expression of *SlWUS*, thus maintaining SAM activity (Figure 5C). We also noticed SlGCN5 may play a role in floral organ development. These findings could potentially shed light on genetic enhancement of tomato plants.

## Data Availability Statement

The original contributions presented in the study are included in the article/Supplementary Material, further inquiries can be directed to the corresponding author/s.

## Author Contributions

BS conceived and designed research, wrote the manuscript, and revised the manuscript. AH and SX conducted experiments. ZY performed data analysis. All authors have read and approved the manuscript.

## Conflict of Interest

The authors declare that the research was conducted in the absence of any commercial or financial relationships that could be construed as a potential conflict of interest.

## Publisher’s Note

All claims expressed in this article are solely those of the authors and do not necessarily represent those of their affiliated organizations, or those of the publisher, the editors and the reviewers. Any product that may be evaluated in this article, or claim that may be made by its manufacturer, is not guaranteed or endorsed by the publisher.

## Footnotes

1
https://solgenomics.net

2
http://wmd3.weigelworld.org./cgi-bin/webapp.cgi

3
https://phytozome-next.jgi.doe.gov/
