# Supplementary material for: Histone Acetyltransferase SlGCN5 Regulates Shoot Meristem and Flower Development in Solanum lycopersicum
Source: Front Plant Sci. 2022 Jan 21;12:805879. doi: 10.3389/fpls.2021.805879 (PMC8814577; doi:10.3389/fpls.2021.805879)
Supplement: Supplementary file 1 [file Data_Sheet_1.PDF]

Table 1. Primers used in this study

**qRT-PCR assays**	
Primer name	Sequence 5’to 3’
ACTIN2-F	CTGGAATGGTGAAGGCTGG
ACTIN2-R	TTGGATACTTCAGAGTGAG
SlACT-F	CAGCAGATGTGGATCTCAAA
SlACT-R	CTGTGGACAATGGAAGGAC
SlGCN5(RT)-F	TCACCCTCACATTCTGCGTC
SlGCN5(RT)-R	CGGAGTCATCTTCGGAGTCG
SlWUS(RT)-F	CCCATGTGAAGATGGTGATG
SlWUS(RT)-R	CTTCCATGCAAATGATTCCA
SlCLV1-qRT-F	TTCTGCTGGATTCCGATTACG
SlCLV1-qRT-R	TCACTCTTTTGGTCAACTTTCA
SlCLV3-qRT-F	AAAGGAAGTTGCTCCTGTGAA
SlCLV3-qRT-R	CCTCTTAGCTCCCAATCAGC
SlGCN5-X1-F	AGGCTGGATGGACTCCTGAT
SlGCN5-X1-R	TCAGGAACATCCCGTGCATC
SlGCN5-X2-F	GTTTCAGGGGCGGTAGTGAA
SlGCN5-X2-R	ACTTCTGGCTGTCCATCACA
SlGCN5-X3-F	GCTTGAGAGAGGCTGGATGG
SlGCN5-X3-R	TGCCAAGTTAGCTTTTTCAGGA
SlADA2a-X1-F	GTCTAAGAGAAAAAGGGCAGTGC
SlADA2a-X1-R	CTGCATATCCTTGATCCTTGGC
SlADA2a-X2-F	GTCTTGTGATGGACAGCCAGA
SlADA2a-X2-R	CAATCTGGTGCCATAGCCCT
SlADA2a-X3-F	GCTTGAGAGAGGCTGGATGG
SlADA2a-X3-R	CCAACTCACTCAACTTTGCCA
SlADA2b-X1-F	TTCAGAGTTAAATGGGTCGCTC
SlADA2b-X1-R	ACCAACAGAGAAGCACTCTATACATAGG
SlADA2b-X2-F	AGTGGCTGTGAAATTGTTTAGGT
SlADA2b-X2-R	AACGCTCATCCTCGCACAAT

**Generating DNA construct**	
Primer name	Sequence 5’to 3’
35S::SlGCN5-GFP-F	GCTACGCGTCTCGAG ATGGACGCTTCGCACTTG
35S::SlGCN5-GFP-R	TCCGGCGCCGGGCCCCTGAATCTTGATGCTAGACTGTAGAC
PYL156-SlGCN5-F	AAGGTTACCGAATTCCGGTGCAGATTCCGACTC
PYL156-SlGCN5-F	CCTTCTAGAGAATTCCTCCATCGTTTGAAACACATAC

***In situ* hybridization**	
Primer name	Sequence 5’ to 3’
SlWUS-F	ATGGAACATCAACACAACATAGAAG
SlWUS-R	TTAGGGGAAAGAGTTGAGAGTAAGT

SlGCN5-F	ATGGACGCTTCGCACTTG
SlGCN5-R	TTCTTCATACTCTCATCC

**Interaction between SlADA2b and SlGCN5**	
Primer name	Sequence 5’to 3’
AD-SlGCN5-F	TGGAGGCCAGTGAATTC ATGGACGCTTCGCACTTG
AD-SlGCN5-R	CACCCGGGTGGAATTCCTACTGAATCTTGATGCTAGACTGT
BD-SlADA2a-F	CATGGAGGCCGAATTCATGGGTCGTTCTCGGGC
BD-SlADA2a-R	CAGGTCGACGGATCCTTATGCTTGAGCCAGGCC
BD-SlADA2b-F	CATGGAGGCCGAATTCATGGGTCGCTCTCGTGG
BD-SlADA2b-R	CAGGTCGACGGATCC TTACAAGGGTGCAACTCCTTT
75-SlGCN5-F	TCTCGAGCTCAAGCTATGGACGCTTCGCACTTG
75-SlGCN5-R	AGATCAGGTGGATCCCTACTGAATCTTGATGCTAGACTGT
76-SlADA2a-F	TCTCGAGCTCAAGCTATGGGTCGTTCTCGGGC
76-SlADA2a-R	AGATCAGGTGGATCC TTATGCTTGAGCCAGGCC
76-SlADA2b-F	TCTCGAGCTCAAGCT ATGGGTCGTTCTCGGGC
76-SlADA2b-R	AGATCAGGTGGATCCTTACAAGGGTGCAACTCCTTT

**amiRNA construct**	
Primer name	Sequence 5’to 3’
SlGCN5miR-s1	GATTACAAACTTGAGTCTTGCTGTCTCTCTTTTGTATTCC
SlGCN5miR-a1	GACAGCAAGACTCAAGTTTGTAATCAAAGAGAATCAATGA
SlGCN5miR*s1	GACAACAAGACTCAACTTTGTATTCACAGGTCGTGATATG
SlGCN5miR*a1	GAATACAAAGTTGAGTCTTGTTGTCTACATATATATTCCT

```
AtGCN5         MDSHSSHLNAANRSRSSQTPSPSHSASASVTSSLHKRKLAATTAANAAASEDHAPPSSSFPPSSFSADTR   70
XP_004248823   ..MDASHLTAPGRSRSSQSPSPSHSASASATSSIHKRKMAS.........DDHAPP...FP.SSFS.DTR   54
XP_025883474   ..MDASHLTAPGRSRSSQSPSPSHSASASATSSIHKRKMAS.........DDHAPP...FP.SSFS.DTR   54
XP_025883475   ..MDASHLTAPGRSRSSQSPSPSHSASASATSSIHKRKMAS.........DDHAPP...FP.SSFS.DTR   54
Consensus           shl a  rsrssq pspshsasas tss hkrk a           dhapp   fp ssfs dtr

AtGCN5         DGALTSNDELESISARGADTDSDPDESEDIVVDDDEDEFAPEQDQDSSIRTFTAARLDSSSGVNGSSRNT   140
XP_004248823   DGALTSNDDLESISARGGGADSDSEDDSEEVGDDDEEDYDAS.....SIRNFTASRLETSVAMVPSGRNT   119
XP_025883474   DGALTSNDDLESISARGGGADSDSEDDSEEVGDDDEEDYDAS.....SIRNFTASRLETSVAMVPSGRNT   119
XP_025883475   DGALTSNDDLESISARGGGADSDSEDDSEEVGDDDEEDYDAS.....SIRNFTASRLETSVAMVPSGRNT   119
Consensus      dgaltsnd lesisarg   dsd       v ddde           sir fta rl  s     s rnt

AtGCN5         KLKTESSTVKLESSDGGKDGGSSVVGTGVSGTVGGSSISGLVPKDESVK.VLAENFQTSGAYIAREEALK   209
XP_004248823   KIKSDN.SVKIESSEIAKEVGTGCSGNVAPPTATG.SVSGAVVKDESMKNIFTENLQTSGAYTAREESLK   187
XP_025883474   KIKSDN.SVKIESSEIAKEVGTGCSGNVAPPTATG.SVSGAVVKDESMKNIFTENLQTSGAYTAREESLK   187
XP_025883475   KIKSDN.SVKIESSEIAKEVGTGCSGNVAPPTATG.SVSGAVVKDESMKNIFTENLQTSGAYTAREESLK   187
Consensus      k k     vk ess   k  g    g     t  g s sg v kdes k    en qtsgay aree lk

AtGCN5         REEQAGRLKFVCYSNDSIDEHMMCLIGLKNIFARQLPNMPKEYIVRLLMDRKHKSVMVLRGNLVVGGITY   279
XP_004248823   REEESGRLKFVCVSNDGVDEHMIWLVGLKNIFARQLPNMPKEYIVRLVMDRNHKSVMVIRRNLVVGGITY   257
XP_025883474   REEESGRLKFVCVSNDGVDEHMIWLVGLKNIFARQLPNMPKEYIVRLVMD....................   237
XP_025883475   REEESGRLKFVCVSNDGVDEHMIWLVGLKNIFARQLPNMPKEYIVRLVMDRNHKSVMVIRRNLVVGGITY   257
Consensus      ree  grlkfvc snd  dehm  l glknifarqlpnmpkeyivrl md

AtGCN5         RPYHSQKFGEIAFCAITADEQVKGYGTRLMNHLKQHARDVDGLTHFLTYADNNAVGYFVKQGFTKEIYLE   349
XP_004248823   RPYTSQKFGEIAFCAITADEQVKGYGTRLMNHLKQHARDVDGLTHFLTYADNNAVGYFVKQGFTKEIYLE   327
XP_025883474   ....SQKFGEIAFCAITADEQVKGYGTRLMNHLKQHARDVDGLTHFLTYADNNAVGYFVKQGFTKEIYLE   303
XP_025883475   RPYTSQKFGEIAFCAITADEQVKGYGTRLMNHLKQHARDVDGLTHFLTYADNNAVGYFVKQGFTKEIYLE   327
Consensus          sqkfgeiafcaitadeqvkgygtrlmnhlkqhardvdglthfltyadnnavgyfvkqgftkeiyle

AtGCN5         KDVWHGFIKDYDGGLLMECKIDPKLPYTDLSSMIRQQRKAIDERIRELSNCQNVYPKIEFLKNEAGIPRK   419
XP_004248823   KERWQGYIKDYDGGILMECKIDPKLPYTDLSTMIRRQRQAIDEKIRELSNCHIVYPGIDFQKKEAGIPKK   397
XP_025883474   KERWQGYIKDYDGGILMECKIDPKLPYTDLSTMIRRQRQAIDEKIRELSNCHIVYPGIDFQKKEAGIPKK   373
XP_025883475   KERWQGYIKDYDGGILMECKIDPKLPYTDLSTMIRRQRQAIDEKIRELSNCHIVYPGIDFQKKEAGIPKK   397
Consensus      k  w g ikdydgg lmeckidpklpytdls mir qr aide irelsnc  vyp i f k eagip k

AtGCN5         IIKVEEIRGLREAGWTPDQWGHTRFKLFNGSADMVTNQKQLNALMRALLKTMQDHADAWPFKEPVDSRDV   489
XP_004248823   AIKVEDIPGLREAGWTPDQYGHSRFKTVN....SLSDQKSLTAFMRSLLKIMHDHPDSWPFKEPVDARDV   463
XP_025883474   AIKVEDIPGLREAGWTPDQYGHSRFKTVN....SLSDQKSLTAFMRSLLKIMHDHPDSWPFKEPVDARDV   439
XP_025883475   AIKVEDIPGLREAGWTPDQYGHSRFKTVN....SLSDQKSLTAFMRSLLKKLTWQS..............   449
Consensus       ikve i glreagwtpdq gh rfk  n        qk l a mr llk

AtGCN5         PDYYDIIKDPIDLKVIAKRVESEQYYVTLDMFVADARRMFNNCRTYNSPDTIYYKCATRLETHFHSKVQA   559
XP_004248823   PDYYDIIKDPIDLKTMSKRVESELYYVTFDMFLADVRRMFANARTYNSPETIYYKCSTRLESHFSNKVQA   533
XP_025883474   PDYYDIIKDPIDLKTMSKRVESELYYVTFDMFLADVRRMFANARTYNSPETIYYKCSTRLESHFSNKVQA   509
XP_025883475   ......................................................................   449
Consensus

AtGCN5         GLQSGAKS                                                                 567
XP_004248823   GLQSSIKI                                                                 541
XP_025883474   GLQSSIKI                                                                 517
XP_025883475   ........                                                                 449
Consensus
```

Fig S1. Alignments of amino aicd sequences of three isoforms of SlGCN5 with AtGCN5. The amino acid sequences of SlGCN5 isoforms were obtained from NCBI (http://www.ncbi.nlm.nih.gov) by BLAST using AtGCN5 as a query. The Identical amino acids are shown in black color, and analogous amino acids are in gray colors.

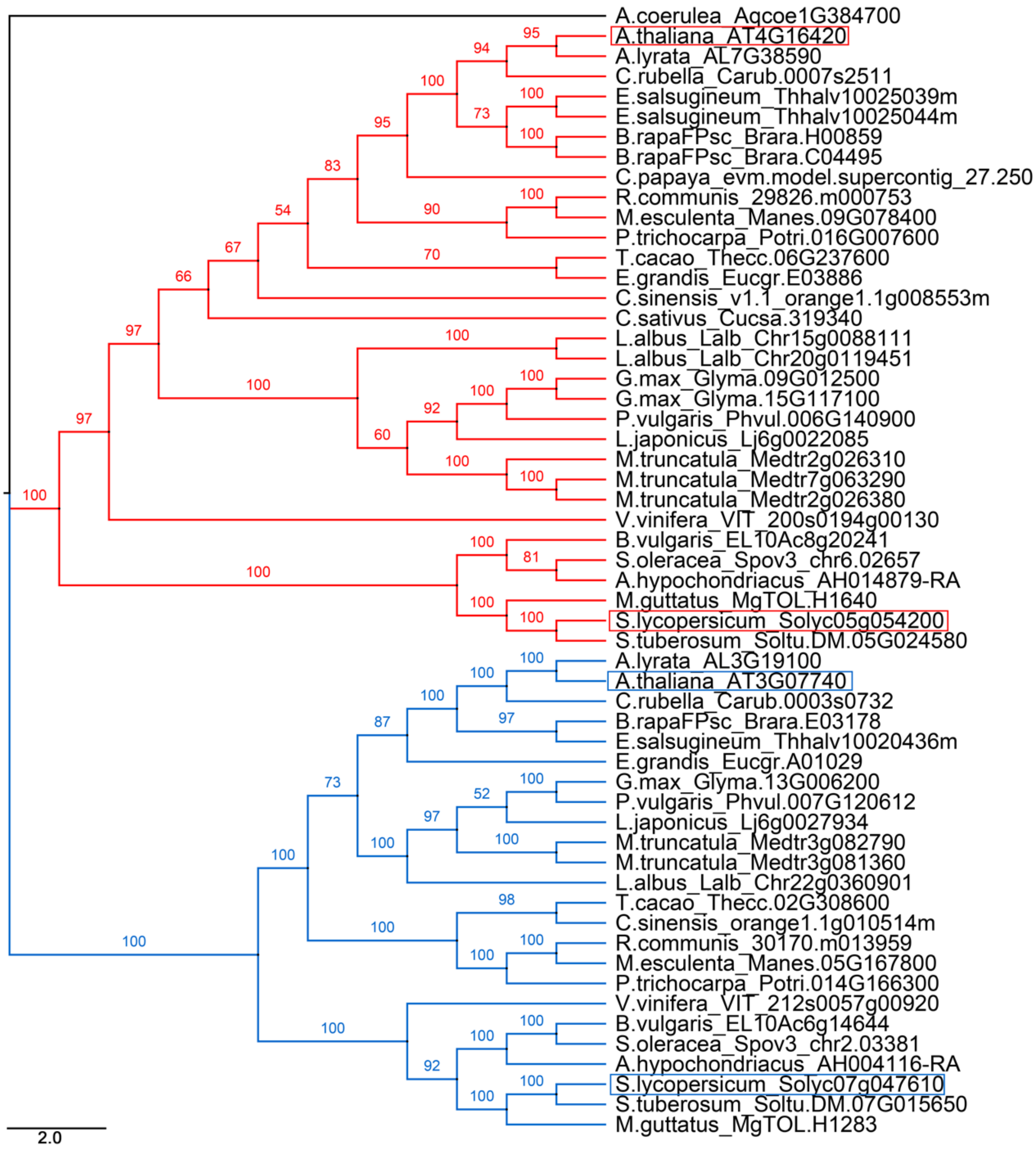

Fig S2. Phylogenetic analysis of *ADA2a* and *ADA2b* in dicot plant species**.** The red box represents *Ada2b* in *Arabidopsis* and *Solanum lycopersicum*, and the blue box represents *Ada2a* in Arabidopsis and *Solanum lycopersicum*.

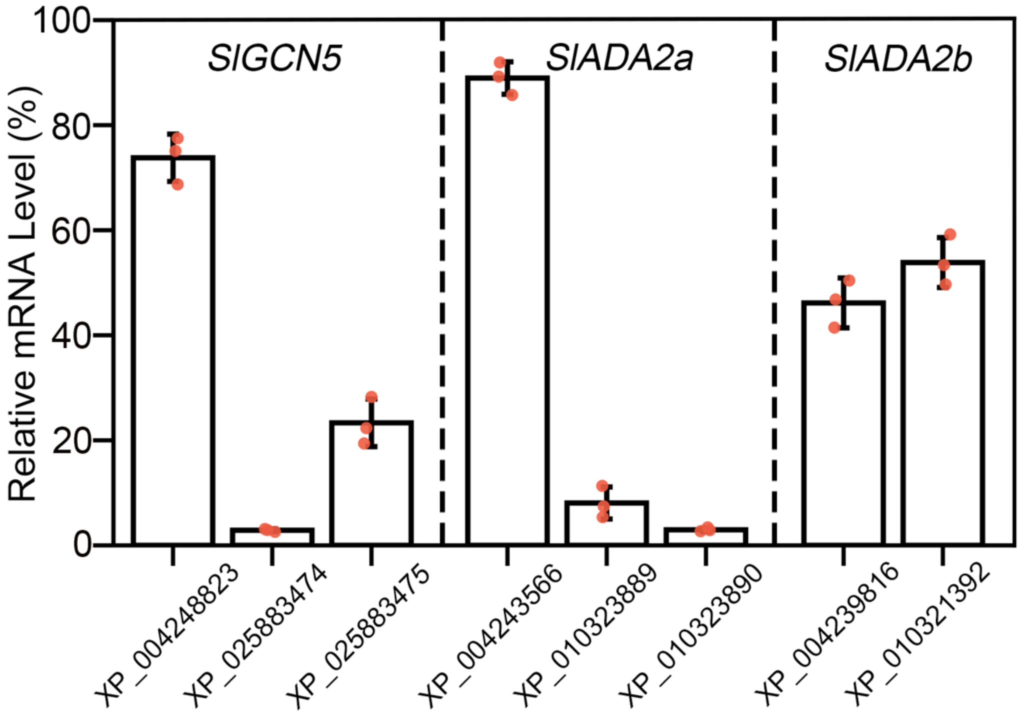

Fig S3. Analysis of *SlGCN5*, *SlADA2a* and *SlADA2b* transcript expression level in tomato inflorescences.

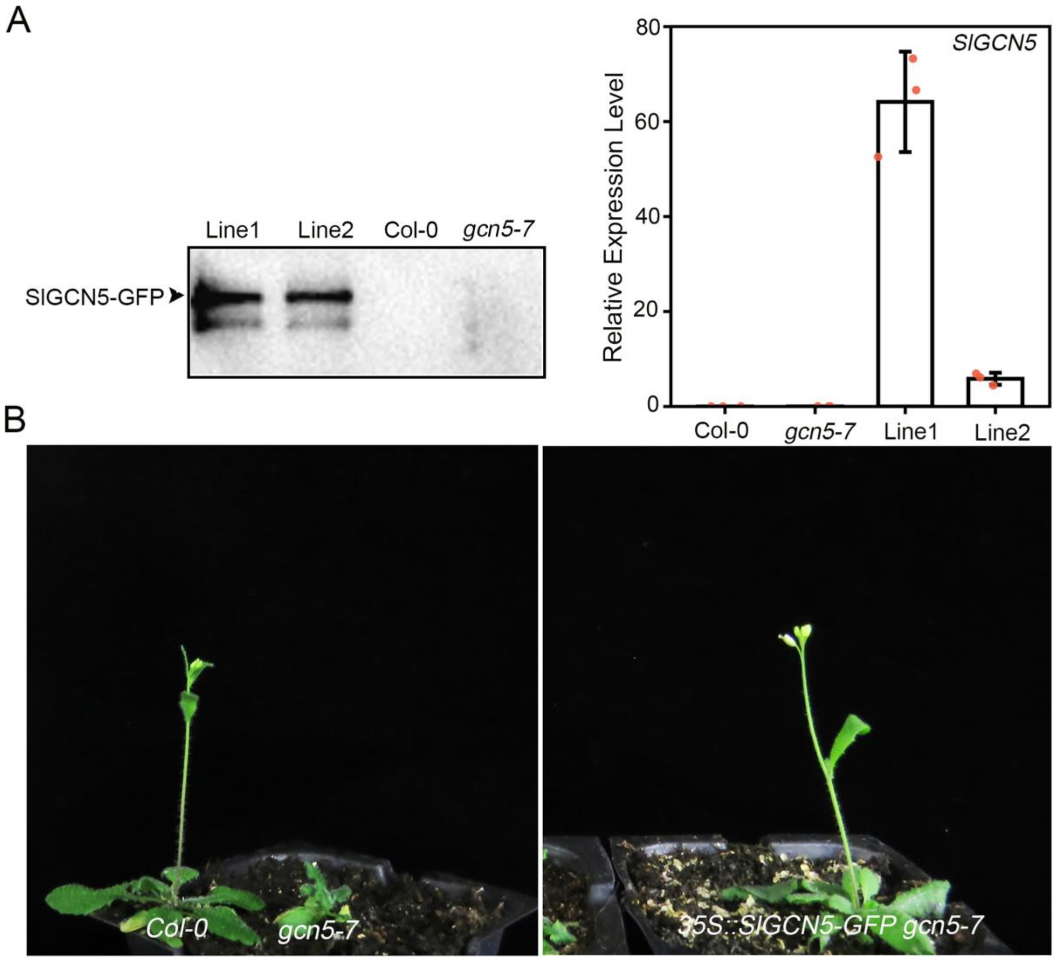

Fig S4. *35S::SlGCN5-GFP gcn5-7* shows fully rescued phenotype. **(A)** Protein and transcript level analysis of SlGCN5 in wild type, *gcn5-7* and *35S::SlGCN5-GFP gcn5-7 Arabidopsis* plants. Error bar represents SD of three biological replicates. **(B)** *35S:SlGCN5-GFP gcn5-7* shows fully rescued phenotype compared to Col-0 wildtype and *gcn5-7* plants.

.

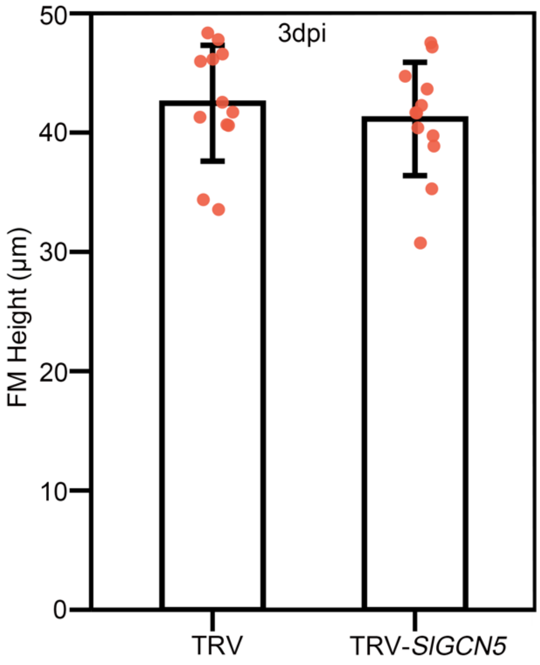

Fig S5. FM height from TRV control and TRV-*SlGCN5* plants at 3 dpi (days post floral initiation). Error bar indicates SD of 12 biological replicates.

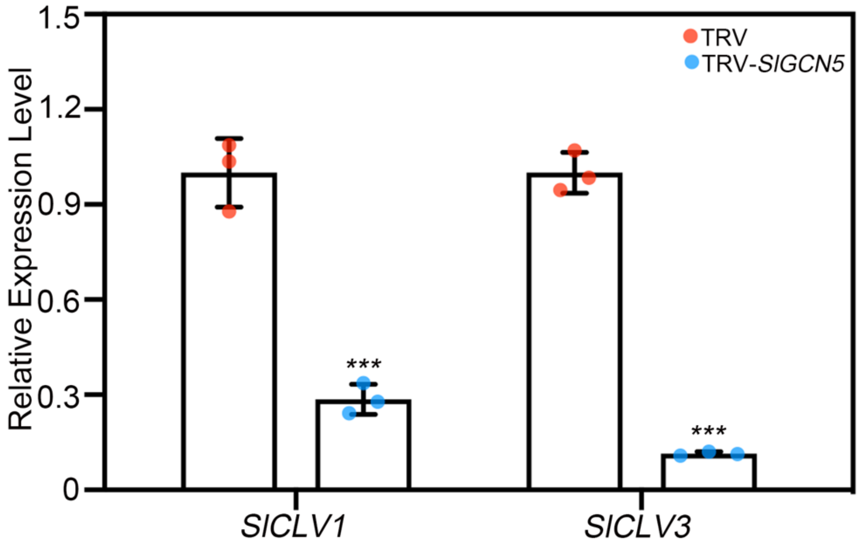

Fig S6. qRT-PCR analysis of *SlCLV1 and SlCLV3* transcripts in TRV control and TRV-*SlGCN5* plants. Error bars represent SD of three biological replicates. Asterisks indicate significant differences (***P < 0.001)
